# A Novel 3D-Printed Negative-Stiffness Lattice Structure with Internal Resonance Characteristics and Tunable Bandgap Properties

**DOI:** 10.3390/ma16247669

**Published:** 2023-12-15

**Authors:** Jiayang Liu, Shu Li

**Affiliations:** School of Aeronautic Science and Engineering, Beihang University, 37 Xueyuan Road, Beijing 100191, China; ljy_bh@buaa.edu.cn

**Keywords:** negative stiffness, lattice structures, bandgap tuning, vibration reduction, transmission spectrum

## Abstract

The bandgap tuning potential offered by negative-stiffness lattice structures, characterized by their unique mechanical properties, represents a promising and burgeoning field. The potential of large deformations in lattice structures to transition between stable configurations is explored in this study. This transformation offers a novel method for modifying the frequency range of elastic wave attenuation, simultaneously absorbing energy and effectively generating diverse bandgap ranges. In this paper, an enhanced lattice structure is introduced, building upon the foundation of the normal negative-stiffness lattice structures. The research examined the behavior of the suggested negative-stiffness lattice structures when subjected to uniaxial compression. This included analyzing the dispersion spectra and bandgaps across different states of deformation. It also delved into the effects of geometric parameter changes on bandgap properties. Furthermore, the findings highlight that the normal negative-stiffness lattice structure demonstrates restricted capabilities in attenuating vibrations. In contrast, notable performance improvements are displayed by the improved negative-stiffness lattice structure, featuring distinct energy band structures and variable bandgap ranges in response to differing deformation states. This highlights the feasibility of bandgap tuning through the deformation of negatively stiffened structures. Finally, the overall metamaterial structure is simulated using a unit cell finite element dynamic model, and its vibration transmission properties and frequency response patterns are analyzed. A fresh perspective on the research and design of negative-stiffness lattice structures, particularly focusing on their bandgap tuning capabilities, is offered in this study.

## 1. Introduction

Lattice structures are gaining attention in numerous fields, attributed to their exceptional qualities, including low density, high stiffness, and superior energy absorption capabilities [1,2,3,4,5]. They can achieve enhanced functional or mechanical properties compared to bulk materials, owing to their geometric flexibility [6]. Their distinctive properties include negative Poisson’s ratio auxetic structures [7], negative stiffness [8], negative compressibility [9], negative thermal expansion coefficients [10], and high stiffness-to-mass ratios [11].

Periodic lattice structures can inhibit elastic wave propagation within certain frequency ranges, called bandgaps [12,13]. They have attracted attention in vibration control applications due to their elastic wave modulation capability. Villeneuve and Piche [14] investigated the bandgap widths and positions of two-dimensional square and hexagonal lattice structures using the plane wave expansion method. They adjusted the bandgap size and position by altering lattice dimensions and material refractive index. Qiu and He [15] suggested a two-dimensional photonic crystal structure consisting of elliptical air holes, which can achieve a broadband complete bandgap, with potential applications for photonic devices. Wang et al. [16] developed a phononic crystal with unidirectional elastic boundary wave propagation by introducing topological changes into the lattice structure. Wagner et al. [17] examined the impact of disorder on the bandgap properties of phononic crystals, discovering that moderate disorder can enhance the size and completeness of the bandgap. Luca et al. [18] conducted experimental investigations to verify the presence of a frequency bandgap in microstructural materials containing resonators. Xiang et al. [19] concentrated on identifying parameters that lead to optimized distributions of local internal resonators, aiming to enhance the vibro-acoustic performance of anti-tetra-chiral auxetic sandwich panels. The incorporation of localized resonators in these anti-tetra-chiral sandwich structures significantly improves sound attenuation, especially under local loading conditions.

Traditional lattice structures with fixed microstructures can only provide fixed-frequency bandgaps, while tunable bandgaps require changes in the lattice structure’s internal dimensions or configurations [14]. As outlined in [20], the contributions of acoustic metamaterials to the domains of sound absorption, insulation, cloaking, and imaging are noteworthy. These materials enable both active and passive tuning, facilitating the development of reconfigurable materials capable of unprecedented manipulation of waves. Thus, reconfigurable lattice structures have gained increased attention, with strain application being one of the most common methods. For instance, Gasparetto and ElSayed [21] investigated adjusting phononic bandgaps in two-dimensional Bloch-periodic lattice structures using deformation. Gao et al. [22] proposed a two-dimensional soft phononic crystal structure based on orthogonal elliptical voids, achieving controllable adjustment of bandgap width and position by modifying the aspect ratio and arrangement of the voids. Pal et al. [23] explored the wave characteristics of hexagonal lattices under large deformation pre-loads, finding that the lattice’s bandgap width and position can be effectively controlled under suitable pre-loads. Furthermore, reconfiguration of lattice structures composed of stimulus-responsive components can be induced through a specific type of stimulus. Casadei et al. [24] introduced a method of constructing tunable acoustic waveguides and phononic metamaterials using a piezoelectric resonator array, achieving tunability of the waveguide and negative refraction properties of the metamaterial by altering the resonator frequency and spacing. Schaeffer et al. [25] studied wave propagation properties in magneto-elastic lattices and found that wave behavior and frequency range can be controlled by adjusting the magnetic field and pre-compression.

To maintain the deformed configuration of reconfigurable lattice materials, it is necessary to sustain the external stimulus that induced the configurational change. Consequently, some studies have focused on large-wave propagation in multi-stable systems composed of negative-stiffness lattice structures (NSLSs). NSLSs, comprising elements with negative stiffness behavior, exhibit superior energy dissipation performance and complete recoverability due to elastic buckling, rather than plastic deformation typical of traditional structures, when subjected to mechanical loads exceeding the threshold.

NSLSs have potential applications in various areas, such as vibration control [26,27,28,29,30,31], wave propagation control [32,33,34,35], energy absorption [8,36,37,38,39,40,41,42,43,44,45], actuation [46,47], energy harvesting [48,49], and deployable structures [50,51,52]. Current research suggests that mechanical metamaterials capable of generating large strains have excellent bandgap tuning capabilities [53,54,55], indicating that the nonlinear behavior of NSLSs makes them an ideal candidate for producing tunable elastic metamaterials. Moreover, NSLS is programmable and repeatable, and can be designed to limit the response at a predetermined level upon impact [56]. Given that the fundamental characteristics of lattice structures are determined by the geometry and stiffness of the unit cell, it is anticipated that each stable configuration will possess a distinct range of bandgap frequencies.

Recent research on energy absorption characteristics and bandgap properties of negative-stiffness lattice structures (NSLSs) has been steadily growing [32,57,58]. In this study, we initially selected a unit cell configuration similar to that described in Ref. [58]. As reported in the literature, this material exhibits characteristics akin to local resonance materials, displaying a low-frequency bandgap in small-amplitude plane waves. However, in contrast to other multi-stable unit materials, changes in configuration corresponding to different stability levels do not substantially alter the material’s wave propagation behavior. To address this limitation, this paper conducts an in-depth investigation of this structure, proposing a new negative-stiffness lattice structure (meta-NSLS) characterized by significant deformability, reconfigurability, multi-directional bandgap tunability, and an extended bandgap tunability range. This paper will provide a detailed description of the geometric shape, deformation process, and mechanical performance of the proposed NSLS under mechanical loads. Furthermore, the study will investigate the NSLS’s ability to regulate the bandgap range upon deformation, primarily through numerical simulation analysis of its band structure and the influence of various parameters on the bandgap in different deformation states.

The paper’s structure is outlined as follows. In Section 2, we propose both normal NSLS and the improved meta-NSLS, and describe the deformation processes of these two types of unit cells. Simultaneously, we investigate the deformation process and compressive behavior of NSLSs with a configuration of 3 × 2 unit cells under quasi-static axial compression given that the geometric constant Q meets the requirements. Section 3 examines and compares the energy band structures of these two structures across various deformation states. Special attention is paid to the analysis of how various geometric parameters impact the formation and size of the bandgap in meta-NSLS. Section 4 conducts a transmittance analysis of these two NSLSs and presents visualizations of their dynamic responses. In conclusion, Section 5 provides a summary of the key discoveries achieved in this study.

## 2. Structural Design

### 2.1. Normal NSLS and Meta-NSLS

**Figure 1:**
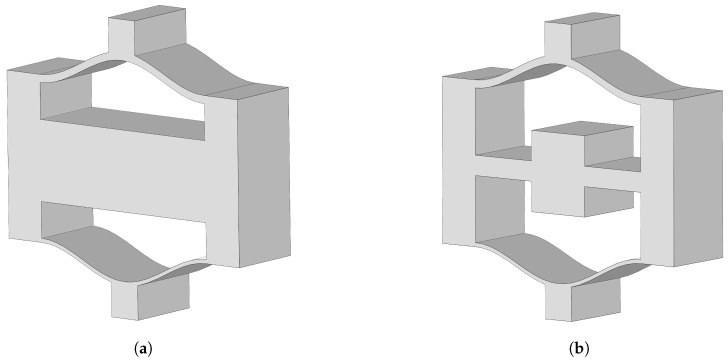
Schematic diagrams of (**a**) the normal NSLS and (**b**) the meta-NSLS.

Figure 1a shows a typical negative-stiffness lattice structure (NSLS) composed of upper and lower layers forming a unit cell. Building on the design concept of local resonant metamaterials, a lattice–beam–core structure as shown in Figure 1b is proposed on the basis of the traditional negative-stiffness lattice structure. The newly proposed structure, named meta-NSLS, differs from the previous lattice structure in that the central beam is modified to be cross-shaped. Drawing from the added periodic design of the internal core and the inherent periodic nature of the lattice structure, it is expected to generate a coupling effect, thereby achieving broadband vibration reduction.

### 2.2. Selection of Geometric Parameters

**Figure 2:**
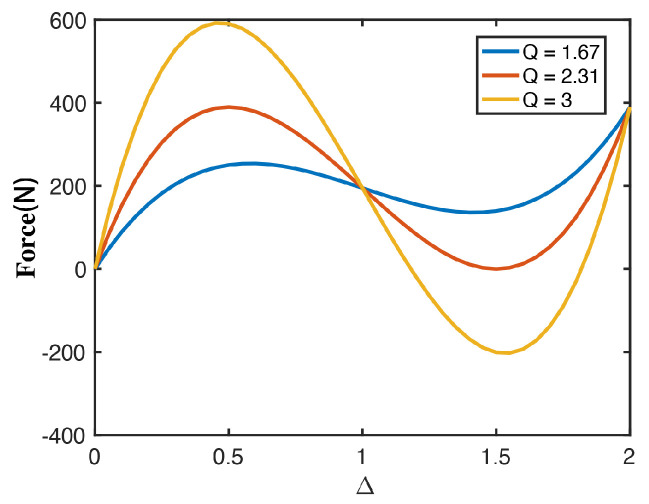
The normalized force against normalized displacement for different values of Q in Equation (2).

As revealed in a previous study [59], the relationship between force and displacement for a curved beam under lateral loads is expressed as follows:

Here is the equation with the quantities in italics:

(1) $$F=\frac{3\pi^{4}Q^{2}}{2}\Delta \left(\Delta -\frac{3}{2}+\sqrt{\frac{1}{4}-\frac{4}{3Q^{2}}}\right)\left(\Delta -\frac{3}{2}-\sqrt{\frac{1}{4}-\frac{4}{3Q^{2}}}\right)$$

where Δ represents the ratio of the vertical displacement d to the apex height, denoted as H, of the beam, and *Q* represents the ratio of the apex height H to the in-plane thickness t of the beam. By varying the geometric constant *Q*, the force–displacement curve plotted by this equation is illustrated in Figure 2. It is evident that, as *Q* increases, the beam displays significant negative stiffness behavior. At the same time, for *Q* > 2.31, the curved beam will exhibit bistability.

The initial shape of the curved beam used in this study can be represented as [55]

(2) $$\omega \left(x\right)=\frac{h}{2}\left[1-\cos\left(2\pi \frac{x}{L}\right)\right]$$

**Figure 3:**
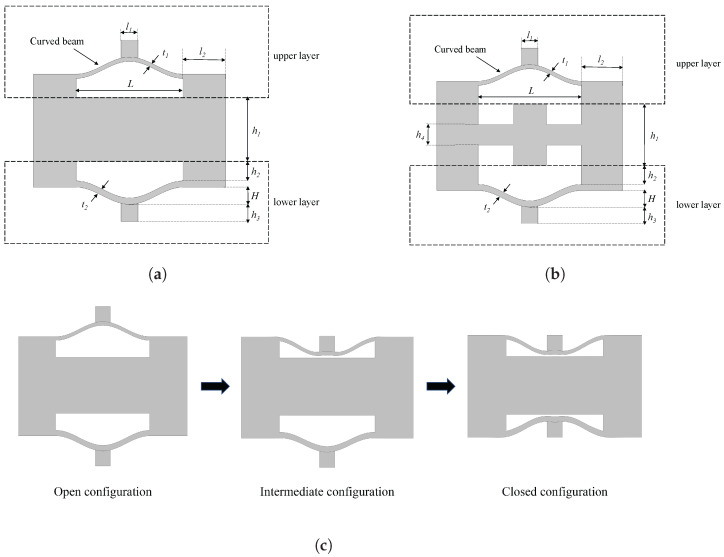
The designed unit cell for NSLS and meta-NSLS consists of (**a**) geometric parameters for the NSLS unit cell, which is composed of upper and lower layers. (**b**) Geometric parameters for the meta-NSLS unit cell, also with upper and lower layers. (**c**) During deformation under uniaxial compressive loads, the NSLS unit cell undergoes three typical deformation states. The open configuration represents the initial state with no displacement, the intermediate configuration occurs when beams in the upper layer buckle, and the closed configuration is reached when the unit cell is compressed into closed state.

Figure 3a,b show the schematic diagram of the unit cell for two different configurations. The most crucial parameters of the model, such as in-plane thickness of the upper curved beam ($t_{1}$), in-plane thickness of the lower curved beam ($t_{2}$), the height of vertex (H), and the span (*L*), are set according to the principles mentioned above.

Table 1 displays the geometric parameters for each component. In order to induce a change in state, a uniaxial load is applied vertically to the top. Taking the normal NSLS as an example (the deformation process of meta-NSLS is similar to it), its deformation process under a vertical load is illustrated in Figure 3c. These three typical deformation states can be referred to as the open configuration, intermediate configuration, and closed configuration.

To ensure the sequence and determinacy of deformation [56], we designed the structure with $t_{1}$ < $t_{2}$. When the unit cell is under compression, the upper beam will bend and buckle first due to the difference in stiffness, and the cell will transform into the intermediate configuration. Then, the lower beam bends and buckles, transforming the cell into the closed configuration. Additionally, we ensured that *H* < $h_{1}$ to prevent interference between the stable state of the curved beam after compression and the central crossbeam. The thickness of both the central beam and side walls is significantly greater than that of the curved beam. Therefore, the central part of the beam and the side walls can be approximated as rigid bodies. The central beam can effectively prevent the cell from expanding horizontally during compression, while the side walls, with relatively higher stiffness, can effectively limit the rotation of the curved beam during compression, resulting in the same buckling mode as that under fixed boundary conditions, which enables the structure to exhibit the snap-through phenomenon and thus possess negative stiffness properties.

In terms of material modeling, a compressible neo-Hookean constitutive model was implemented to capture the mechanical behavior accurately. Then, the effects of the two proposed designs on the tunable bandgap were investigated. Therefore, we aimed to have controllable deformation of the unit cell during compression. To achieve this, we used thermoplastic polyurethane (TPU) material [57]. TPU is a type of thermoplastic elastomer with many properties, including elasticity, transparency, and abrasion. This material is known for its high tensile strength and elongation, as well as its ability to return to its original shape after stretching. These properties make TPU less prone to permanent deformation under normal conditions. The material properties of TPU are listed in Table 2.

### 2.3. Compression Performance Analysis

To ensure that the unit cell can exhibit the three states shown in Figure 3c during the compression process, we modeled the NSLS using the geometric parameters selected in Table 2 and explored the deformation performance of the structure. Numerical simulations were performed using COMSOL Multiphysics 5.5 to observe the deformation states and determine the ability of the unit cell to deform in a controlled manner when compressed.

**Figure 4:**
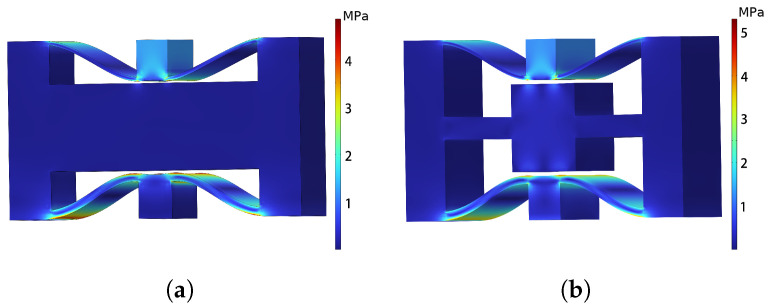
The stress distribution of (**a**) normal NSLS; (**b**) meta-NSLS when it is compressed to the third state.

Firstly, compression simulations were conducted for both normal NSLS and meta-NSLS unit cells, with the results shown in Figure 4a,b. The difference in compressive performance between the two is minimal; both exhibit maximum internal stress when the unit cell is compressed to a closed state. Large stresses are generated at both ends of the curved beams, with the maximum stress for NSLS being 4.85 MPa and for meta-NSLS being 5.14 MPa, both of which are lower than the yield stress of the TPU material (8.6 MPa). The excellent energy absorption characteristics of normal NSLS and meta-NSLS are provided by the curved beams, and, since no modifications are made to the curved beam portion in the configuration, their deformation states and stress distributions are quite similar.

**Figure 5:**
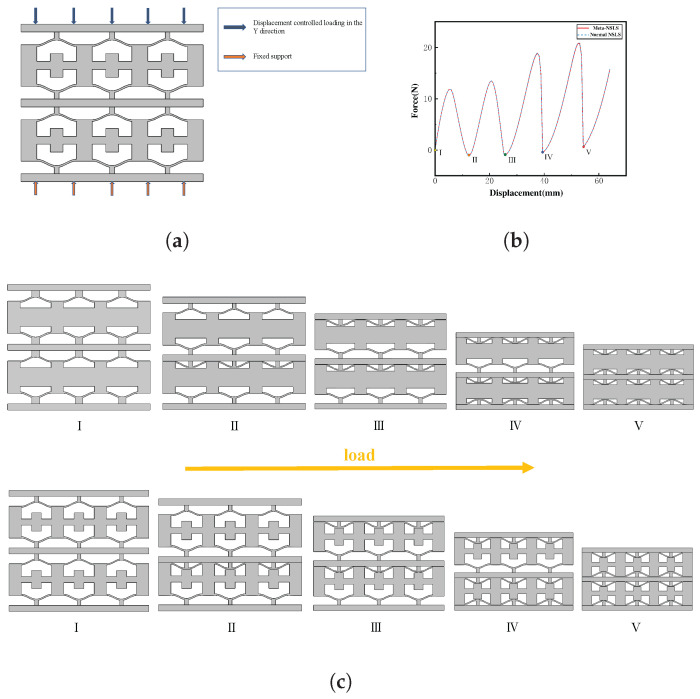
(**a**) The deformationprocess of NSLSs with 3 × 2 unit cells during quasi-static uniaxial compression was simulated by finite element analysis. (**b**) The meta-NSLSs produced the red line results, while the normal NSLSs produced the blue dotted line results, as shown in the force–displacement curves obtained from the simulation. Several special deformation points are also marked with different colors. Due to the similarity in mechanical performance, the two curves almost completely overlap. (**c**) The simulated deformation process obtained under quasi-static uniaxial compression. As the loading continues, the layers with thinner curved beams transform from one state to another first, until all layers have transitioned to the third state, and the loading process ends.

After verifying that the stress in the unit cells satisfies the material requirements, we proceeded to analyze their deformation characteristics. Due to the similarity in compressive performance between NSLS and meta-NSLS, we use NSLSs as an example for the description. As is shown in Figure 5a, NSLSs are formed by the periodic arrangement of NSLS in space. To predict their performance under vertical compression loads, finite element analysis is performed in COMSOL Multiphysics 5.5. Contact pairs are set up in the Solid Mechanics module, and geometric nonlinearity is considered due to the snap-through behavior of the structure. Roller supports are used to support the walls of each vertical unit cell, a specified displacement-controlled load is applied along the negative y-axis on the top surface, and fixed support is provided at the bottom. To streamline the calculations, the physics control network function within the mesh settings was employed for the subdivision of the triangular network into regular-sized segments. Given that the material behaves within a linear elastic regime throughout the strain range under investigation in this study, a linear elastic material model was chosen. The relevant material parameters were then defined within the Solid Mechanics module. In order to simulate quasi-static loading and to capture the full deformation process, displacement-assisted scanning is used in the steady-state investigation. Figure 5b displays the force–displacement curve derived from the simulation, depicting the outcomes for normal NSLSs and meta-NSLSs by the blue dotted and red solid lines, respectively. The results indicate that both configurations can achieve multiple stable states during the compression process with the given geometric parameters, and this multi-stability capability is provided by the curved beams, causing their force and displacement curves to almost overlap.

The deformation characteristics associated with specific force–displacement relationship points projected from the finite element analysis are illustrated in Figure 5c, with different colors representing the locations on the force–displacement curve for different deformation states. For multi-layered structures, as the compression displacement increases, one layer reaches the maximum force at the elastic deformation stage, indicating that the deformation mechanism is mainly composed of bending and compression during the initial stage of deformation. The deformation enters the elastic instability phase when the load on the curved beams reaches the buckling load and snap-through occurs. The slope of the force–displacement graph becomes negative. Each area of negative rigidity results from a series of curved beams changing from one first-mode bending shape to another.

Note that, in compression deformation, the thinner beams in the model require less energy to deform, resulting in the first and third thinner-beam layers deforming before the second and fourth thicker-beam layers. This phenomenon is reflected in Figure 5b, which displays two small and two large peaks. The number of peaks corresponds to the number of layers, while the size of the peaks correlates to the stiffness of the curved beams. In practice, the sequence of layer buckling is affected by the relative defects or weaknesses in one or more beams. This leads to their buckling under slightly smaller compression loads than other layers. The appearance of the fifth positive stiffness region is due to the full compaction of the structure; the compression load is then distributed to the columns on both sides.

## 3. Bandgap Analysis

In this section, three typical deformation states shown in Figure 3c are analyzed due to the different deformation states of the two configurations during the loading process. The manipulation ability of the elastic waves differs among these three states due to the differences in geometric configurations. In order to investigate the functions of these three states in controlling elastic waves, the dispersion analysis method is employed to numerically investigate the band structures of the elastic waves. Under standard temperature conditions, the structure’s elastic modulus, density, and Poisson’s ratio remain constant. To investigate the effects of various deformation states on dispersion regulation during the deformation process, three different configurations of normal and meta-NSLS structures were subject to a series of numerical simulations. Dispersion analysis is performed by implementing the finite element method within the Solid Mechanics module of COMSOL Multiphysics 5.5, and the dispersion relations and transmission spectra of normal and meta-NSLS in the same configuration are compared. Effects of periodic layout, structural parameters, and structural dissipation factor on bandgap properties are also investigated. In this context, Bloch’s theorem is used to analyze the propagation of free waves in mechanical metamaterials.

### 3.1. Dispersion Spectra and Bandgaps

#### 3.1.1. Open Configuration

The dispersion curve in the open state would depict the behavior of waves in the unit cell without any deformation or external forces applied. It represents the baseline dispersion characteristics of the system.

**Figure 6:**
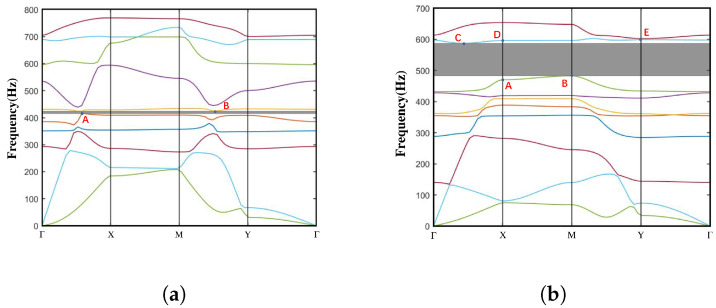
Dispersion curve in the open state of the (**a**) normal NSLS and (**b**) meta-NSLS. Several typical eigenmodes of the normal NSLS (Frequencies A–B in Figure 6a) and meta-NSLS (Frequencies A–E in Figure 6b) are captured at the bandgap edges and visualized in Figure 7 and Figure 8 for deeper discussion on the bandgap mechanism. There are ten branches in the energy band structure curve we calculated, the first branch is the acoustic transverse branch, the second branch is the acoustic longitudinal branch, and the other branches are optical branches, which originate from local resonances within the unit cell and the back-folding (Bragg reflection) of the lower acoustic branches. For better distinction, different dispersion curves are represented by different colors and the bandgap range is shaded in grey.

**Figure A1:**
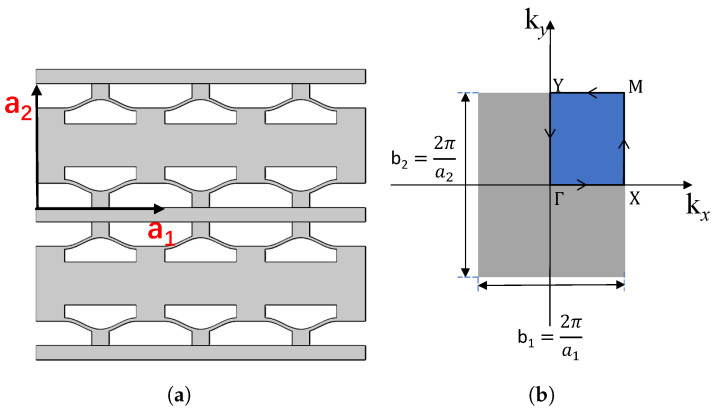
Types of lattices: (**a**) direct lattice (all unit cells are assumed in the open configuration in Figure 3c). (**b**) Reciprocal lattice. The first Brillouin zone is represented by the entire gray area enclosed in a rectangle, and the irreducible Brillouin zone (IBZ) is represented by the blue region labeled as ΓXMY.

Based on the Bloch–Floquet theory, the dispersion spectra of both normal NSLS and meta-NSLS structures are systematically investigated by sweeping the Bloch wave vector along the irreducible Brillouin zone (as illustrated in Figure A1b). For a more comprehensive understanding of the dispersion analysis, additional details can be found in Appendix A. Figure 6a,b depict the dispersion curves of normal NSLS and meta-NSLS structures in the open configuration, respectively, highlighting their different characteristics and performance in the same configuration.

To calculate the bandgap width, the normalized bandgap width is introduced,

(3) $$\;\mathrm{Normalized}\;\mathrm{width}\;=\frac{f_{u}-f_{l}}{\left(f_{u}+f_{1}\right)/2}$$

where $f_{u}$ and $f_{l}$ represent the frequencies of the upper and lower bandgap edges, respectively. Usually, the band structure of a unit cell consists of numerous branches. In this paper, the energy band structure curve consists of 10 branches.

The normal NSLS exhibits a distinct full bandgap between the fifth and sixth branches, with a bandgap frequency range of 415–424 Hz (normalized bandgap width of 0.021). Meta-NSLS generates a complete bandgap between the eight and ninth branches, with a bandgap frequency range of 482–587 Hz (normalized bandgap width of 0.196). It is evident that, although the bandgap of the normal NSLS starts at a lower frequency (415 Hz), the normalized bandgap width of the meta-NSLS is nine times larger than that of the normal NSLS (from 0.021 to 0.196), indicating superior vibration damping properties. Additionally, the finite element calculations in the second chapter clearly demonstrate that, although the two structures differ, their stiffness can be effectively neglected.

**Figure 7:**
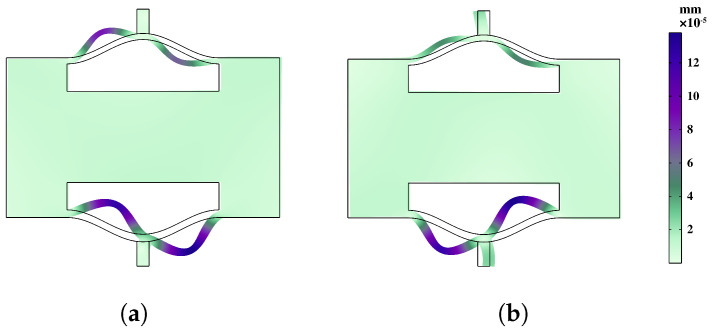
Eigenmodes of the normal NSLS unit cell at (**a**) frequency A; (**b**) frequency B.

**Figure 8:**
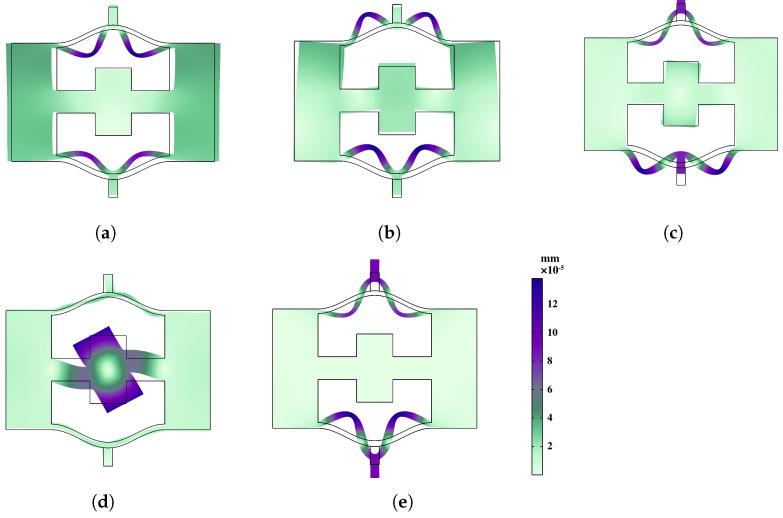
Eigenmodes of the meta-NSLS unit cell. Modes (**a**–**e**) correspond to frequencies A to E in dispersion curves.

In order to conduct a more in-depth discussion on the bandgap mechanism and explain the reasons for the bandgap, we focus on the bandgap edge frequencies represented as A–B in Figure 6a and A–E in Figure 6b. These specific frequencies provide insights into the behavior of the system at the edges of the bandgap. To visualize the corresponding eigenmodes, we examine the displacement fields captured in Figure 7 and Figure 8. These figures shed light on the spatial distribution of the displacements associated with the selected eigenmodes.

Figure 7 displays the bandgap edge frequencies of the fifth and sixth branches in the normal NSLS. It is evident that, in these two states, the upper and lower curved beams of the unit undergo noticeable offset movements. Moreover, due to the disparity in stiffness resulting from the different thicknesses of the upper and lower curved beams, the swing amplitude of the lower curved beam exceeds that of the upper curved beam.

In comparison, the coupling and hybridization effects in meta-NSLS are more pronounced. In addition to the local resonance motion observed in the upper and lower curved beams at points A and E in Figure 6b (Figure 8a,e), there is a distinct local resonance involving the intermediate mass at point D (Figure 8d). By examining Figure 8b–c, it becomes evident that points B and C, located at the edges of the bandgap, exhibit not only the vibration of the curved beam but also the movement of the intermediate mass. These two local resonances are effectively coupled with the oscillations of the upper and lower curved beams through the intermediate mass, giving rise to the formation of an energy bandgap.

Through this analysis, it becomes apparent that the meta-NSLS structure exhibits enhanced coupling and hybridization effects, resulting in a more pronounced bandgap mechanism.

#### 3.1.2. Intermediate Configuration

**Figure 9:**
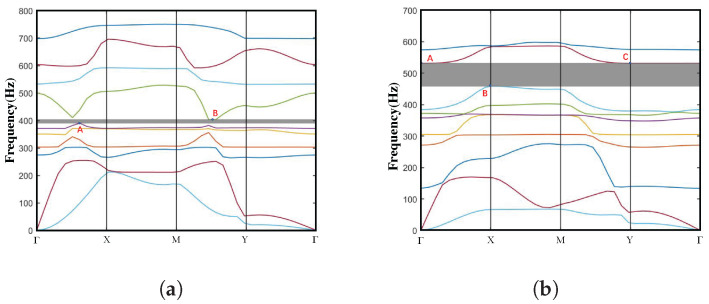
Dispersion curve in the intermediate state of the (**a**) normal NSLS and (**b**) meta-NSLS. To illustrate the physical mechanisms of the bandgaps, two eigenmodes of the normal NSLS at two selected boundary frequencies (Frequencies A–B in Figure 9a) and three eigenmodes of meta-NSLS at three selected boundary frequencies (Frequencies A–C in Figure 9b) are captured and visualized in Figure 10. Same as Figure 6, different dispersion curves are represented by different colors and the bandgap range is shaded in grey.

To calculate the dispersion curves for the latter two stable states, a step-by-step procedure is followed. Firstly, the complete compressive deformation process of the unit cell is obtained via steady-state calculations using COMSOL Multiphysics 5.5. The resulting solution is then utilized to remesh and reconstruct the geometry of the deformed unit cell. This remeshed geometry is subsequently employed as an input object for further analysis. Next, following the methodology described in the Appendix A, the band structure is calculated using the Floquet–Bloch theory. Then, the dispersion curves of the two structures in the intermediate state can be obtained, as shown in Figure 9.

**Figure 10:**
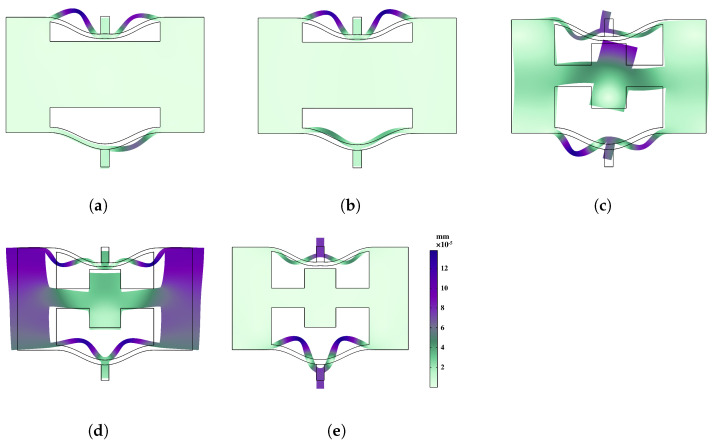
Eigenmodes of the meta-NSLS unit cell. Modes (**a**–**e**) correspond to frequencies A to E in dispersion curves.

In a similar analysis to the previous open state, the normal NSLS structure exhibits a complete bandgap between the sixth and seventh branches, with a bandgap frequency range of 390–403 Hz (normalized bandgap width of 0.032). Meta-NSLS structure produces a complete bandgap between the eight and ninth branches, spanning a frequency range of 458–531 Hz (normalized bandgap width of 0.147). In the immediate state, meta-NSLS structure maintains a normalized bandgap width that is 4.6 times larger than that of the normal NSLS structure, indicating superior damping performance. Additionally, Figure 10 illustrates several typical frequency shift fields at the bandgap edges.

Figure 10a,b correspond to the deformation states of points A and B in Figure 9a, while Figure 10c–e correspond to points A, B, and C in Figure 9b. By analyzing the changes in the displacement field, it becomes evident that the bandgap formation in the second state of normal NSLS is primarily attributed to the local resonance of the upper and lower curved beams. Conversely, in meta-NSLS, the presence of the intermediate mass introduces a joint effect of local resonance and curved beam vibration, resulting in a significantly wider bandgap compared to normal NSLS.

#### 3.1.3. Closed Configuration

**Figure 11:**
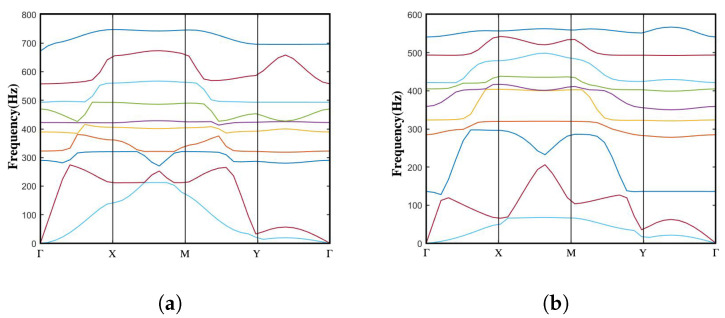
Dispersion curve in the intermediate state of the (**a**) normal NSLS and (**b**) meta-NSLS. Same as Figure 6, different dispersion curves are represented by different colors.

Continuing from the intermediate configuration, the unit cell is further compressed to achieve the closed configuration. The dispersion curves of the two structures in this third-order state are illustrated in Figure 11.

In the third-order state, neither of the two structures exhibits the characteristics of a complete bandgap. As the unit cell undergoes continuous compression, the local resonance of the middle block becomes more restricted. Additionally, the effective stiffness of the upper and lower curved beams undergoes significant changes. Consequently, the bandgap that was present in previous states completely disappears at this stage.

It is worth noting that the disappearance of the bandgap highlights the intricate relationship between the structural deformation and the bandgap formation, emphasizing the importance of understanding the dynamic behavior of the system at different compression levels.

As expected, the dispersion diagrams of the two unit cells in the three states exhibit distinct differences. This phenomenon can be attributed to the significant vertical deformation experienced by the unit cell, which directly affects the lattice constant. Furthermore, the deformation of the structure primarily involves the bending and buckling of the upper and lower curved beams during the compression process, leading to considerable variations in the effective stiffness of the unit cell.

It is noteworthy that the evolution of the bandgap is closely related to the variations in the effective stiffness. As the structure undergoes compression, specific dispersion modes associated with the bandgap experience movement and shifting due to the changing stiffness. This dynamic behavior ultimately leads to the formation, tuning, or broadening of the bandgap frequency range. By applying a compressive load and deforming the metamaterial, it becomes possible to achieve desired modifications in the bandgap properties.

This observation highlights the potential for actively controlling and manipulating the bandgap characteristics of metamaterials through mechanical deformation. Such tunability opens up opportunities for designing novel devices and systems with tailored bandgap properties for various applications.

By comparing the normalized bandgap widths of normal NSLS and meta-NSLS in the same state, a clear distinction emerges. Meta-NSLS, leveraging the phenomenon of local resonance, exhibits a significantly larger bandgap range compared to normal NSLS. This means that, if the criterion for a desired bandgap is increased or if the unit cell is engineered in a way that restricts the motion of the upper and lower curved beams, normal NSLS may lose its effectiveness in providing robust bandgap isolation or offer limited vibration isolation value.

Consequently, the introduction of the new configuration of meta-NSLS holds great value. Its enhanced bandgap width and improved damping performance make it a promising candidate for applications requiring effective vibration control. However, it is important to note that meta-NSLS loses its ability to provide a bandgap in the closed state. Therefore, investigating the impact of geometric parameters becomes crucial in understanding the behavior of the bandgap.

### 3.2. Parametrical Analysis and Discussion

In the preceding analysis, it was observed that meta-NSLS exhibits superior bandgap capabilities compared to NSLS. However, the ability to provide bandgaps is lacking in the third configuration. Therefore, the influence of geometric parameters on bandgap generation becomes particularly crucial. Additionally, it is evident that the central block contributes significantly more to bandgap generation than the upper and lower curved beams. Furthermore, as mentioned in the second paragraph, the geometric properties of the upper and lower curved beams must also satisfy the special physical requirement of bistability. Hence, the geometric parameters of the upper and lower curved beams are not varied, and the focus is primarily on investigating the impact of the size and material of the central block and the connecting narrow beam on the bandgap range.

Moreover, when studying the effects of parameter variations on the structure, a higher criterion for bandgap values is employed. This criterion ensures that the distance between the minimum value of two adjacent bands and the maximum value of the gap exceeds a certain threshold. This approach helps to eliminate the influence of small gaps resulting from resonances in the upper and lower curved beams and mitigates their impact on the research findings.

**Figure 12:**
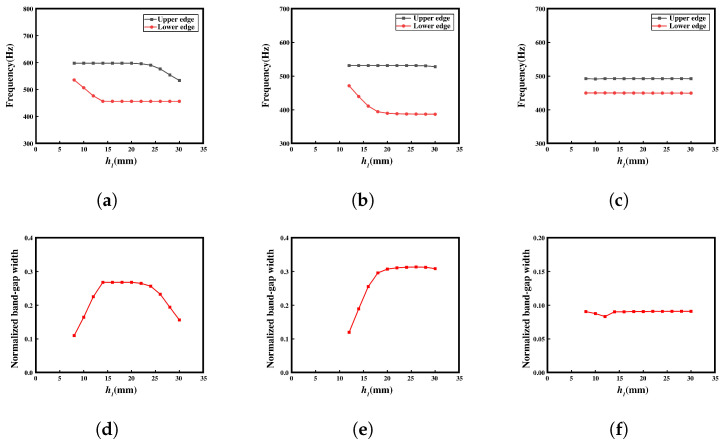
(**a**–**c**) Upper and lower edges of the complete bandgap in three different configurations with varying $h_{1}$ in meta-NSLS. (**d**–**f**) Normalized bandgap width for the three different configurations with varying $h_{1}$ in meta-NSLS.

In Figure 12, the influence of the width $h_{1}$ of the middle block on the band structure is investigated, with the connecting narrow side $h_{4}$ modified to 4 mm to provide a larger design parameter space for $h_{1}$. In the first configuration, the bandgap range exhibits an increasing-then-decreasing trend with varying $h_{1}$. Specifically, when $h_{1}$ is approximately 12 mm, the relative gap width reaches its maximum value, indicating the optimal bandgap capability. In the second configuration, the initial frequency of the bandgap decreases as the mass of the middle resonant block increases, resulting in a downward shift of the bandgap towards lower frequencies. As the ending frequency shows minimal variation, the relative bandgap width continuously increases with the increase in $h_{1}$, leading to a broader bandgap. After $h_{1}$ exceeds 20 mm, the growth in the relative bandgap width becomes relatively slow, approaching a plateau.

Remarkably, the third configuration exhibits a distinct bandgap range, in contrast to the previous results. This observation suggests that the reduction in $h_{4}$ significantly influences the generation of the bandgap. However, with varying $h_{1}$, the starting and ending frequencies of the bandgap remain relatively unchanged, indicating that $h_{1}$ has limited impact on these frequency characteristics.

**Figure 13:**
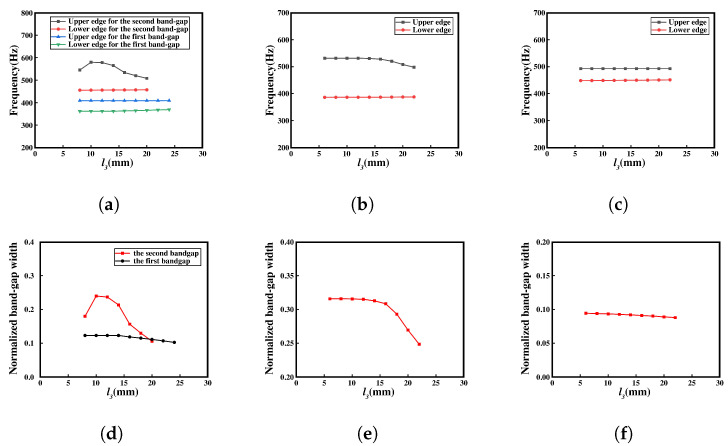
(**a**–**c**) Upper and lower edges of the complete bandgap in three different configurations with varying $l_{3}$ in meta-NSLS. (**d**–**f**) Normalized bandgap width for the three different configurations with varying $l_{3}$ in meta-NSLS.

The effect of the length of the central block ($l_{3}$) on the bandgap was then analyzed, as shown in Figure 13. In the first configuration, two complete bandgaps are observed. The bandgap exhibits a relatively gradual change at low frequencies, while the upper and lower frequency ranges remain relatively stable. In the high-frequency region, the bandgap initially increases and then decreases. The maximum relative bandgap width is achieved when $l_{3}$ is 10 mm. In the second configuration, the relative bandgap width decreases as $l_{3}$ increases. However, in the third configuration, the relative bandgap width remains relatively constant. This is because, with $h_{1}$ set to 30 mm, the local resonance capability of the central block is significantly constrained. Therefore, varying only the value of $l_{3}$ has minimal impact on the bandgap in this configuration.

**Figure 14:**
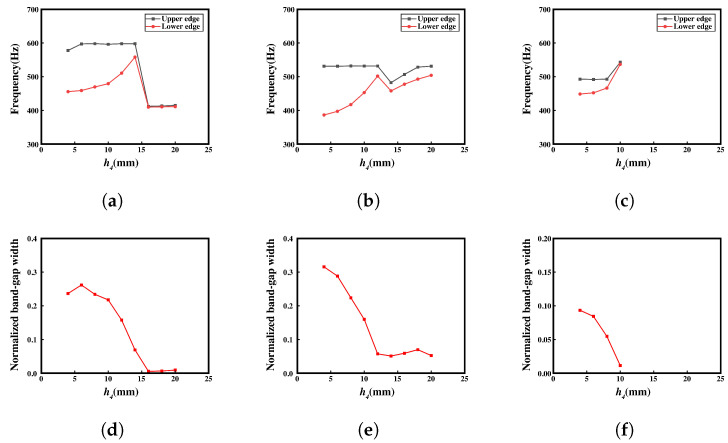
(**a**–**c**) Upper and lower edges of the complete bandgap in three different configurations with varying $h_{4}$ in meta-NSLS. (**d**–**f**) Normalized bandgap width for the three different configurations with varying $h_{4}$ in meta-NSLS. The red lines represent the trend of normalized bandgap width with varying h4 across three different configurations.

Figure 14 shows the impact of the narrow beam $h_{4}$ on the bandgap range of the structure. Previous analysis has also shown that the disappearance of the bandgap is directly related to the size of $h_{4}$. In the first configuration, as $h_{4}$ increases, the relative bandgap width exhibits a linear decreasing trend. When $h_{4}$ exceeds 14 mm, the bandgap experiences a significant drop, almost reducing to zero, indicating a loss in bandgap capability. This can be attributed to the strong local resonance capability of the connected narrow edge when it is thin or significantly narrower than the central block. However, when the narrow edge thickness surpasses a certain critical value, the local resonance capability of the central block becomes severely limited, leading to the loss in bandgap capability.

Similarly, in the second configuration, as $h_{4}$ exceeds 12 mm, the provided bandgap range significantly decreases. This phenomenon has the most pronounced impact on the third configuration, which has a weaker bandgap capability. Once $h_{4}$ exceeds 10 mm, the bandgap completely disappears. Furthermore, in the third configuration, the relative bandgap value is noticeably smaller compared to the first two configurations. The relative bandgap value continues to decrease as $h_{4}$ increases.

Remarkably, the effect of these parameters on the structure’s bandgap is immense as it can guide the design and optimization of the meta-NSLS for desired bandgap characteristics. These findings highlight the significance of geometric parameters and material selection in achieving desired bandgap ranges while considering the constraints imposed by the specific physical properties, such as the requirement for bistability in the upper and lower curved beams.

This section has omitted the study of the influence of material differences between the central and base parts on the bandgap. Drawing from previous research on the phenomenon of local resonance, it is evident that, when the Young’s modulus and density of the central mass part are greater than those of the base part, it is more conducive to the generation of local resonance phenomena. However, considering the primary focus of this paper on the structural enhancements transitioning from the normal NSLS to meta-NSLS, the impact of materials on the bandgap is not a key aspect of this study. Additionally, considering the practicality in manufacturing, ensuring material uniformity between the base and central mass parts is more advantageous for the real-world implementation of 3D printing technology in meta-NSLS production.

The research conducted on the key parameters, namely $l_{3}$, $h_{1}$, and $h_{4}$, has revealed that these variables exert varying degrees of influence on the bandgap range and relative bandgap width in different configurations. The conclusions drawn in this section are consistent with the research results presented in Section 3.1. For the subsequent investigation of vibration transmission in both normal NSLS and meta-NSLS, we have opted for geometric dimensions ($l_{3}$ = 12 mm, $h_{1}$ = 20 mm, and $h_{4}$ = 5 mm) that demonstrate excellent bandgap performance across the three configurations. This selection not only facilitates the visualization of the vibration transmission section but also enables a more comprehensive analysis of the correspondence between the bandgap range and vibration transmission.

## 4. Vibration Transmission

As band structure analysis is derived from infinite periodic structures, this section employs finite periodic structures for vibration testing to validate the findings of the bandgap investigations. This approach ensures a more comprehensive assessment of the structural behavior in finite periodic systems, further enhancing the robustness of the conclusions drawn from the bandgap analysis.

In this part, 9 × 1 unit models were established for both normal NSLS and meta-NSLS. Subsequently, numerical simulations were conducted on these models within the same frequency range. The primary objective was to study the wave propagation characteristics within finite lattice structures and evaluate the vibration damping performance of the two models when subjected to harmonic excitation.

**Figure 15:**
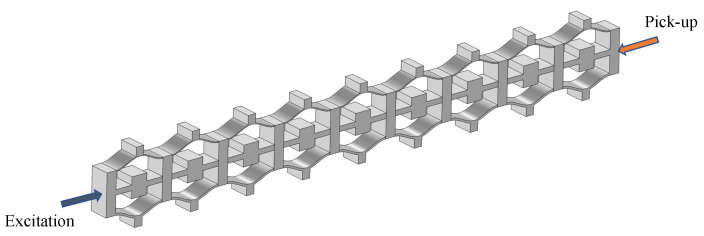
Schematic diagram of the frequency response calculation for meta-NSLSs composed of 9 × 1 unit cells.

The assessment of vibration damping performance involved a detailed analysis of the transmission response under harmonic excitation conditions. Vibration frequency response can be calculated as shown in Figure 15; the vibration transmission, denoted as $\;V_{T}$, was defined using the following equation:

(4) $$V_{T}=20\log_{10}\left(\frac{|A_{\mathrm{out}\;}|}{|A_{\mathrm{in}\;}|}\right)$$

where $A_{\mathrm{out}}$ represents the amplitude of the response recorded at the right center point, while $A_{\mathrm{in}}$ corresponds to the amplitude of the excitation applied at the left end center point.

This procedure involved exciting both normal NSLS and meta-NSLS models with harmonic displacements at varying frequencies; the frequency range considered extended from 10 to 700 Hz. Various frequency harmonic displacements were applied at the left-center point of the sample, and the wave amplitudes were recorded at the other end. By systematically scanning the excitation frequency range and calculating $\;V_{T}$, a comprehensive transmission spectrum was obtained. This spectrum provides valuable insights into the vibration characteristics and damping performance of the structures under investigation.

In summary, the adoption of finite periodic structures for vibration testing adds an important dimension to the study, further enhancing the applicability and robustness of the research findings.

**Figure 16:**
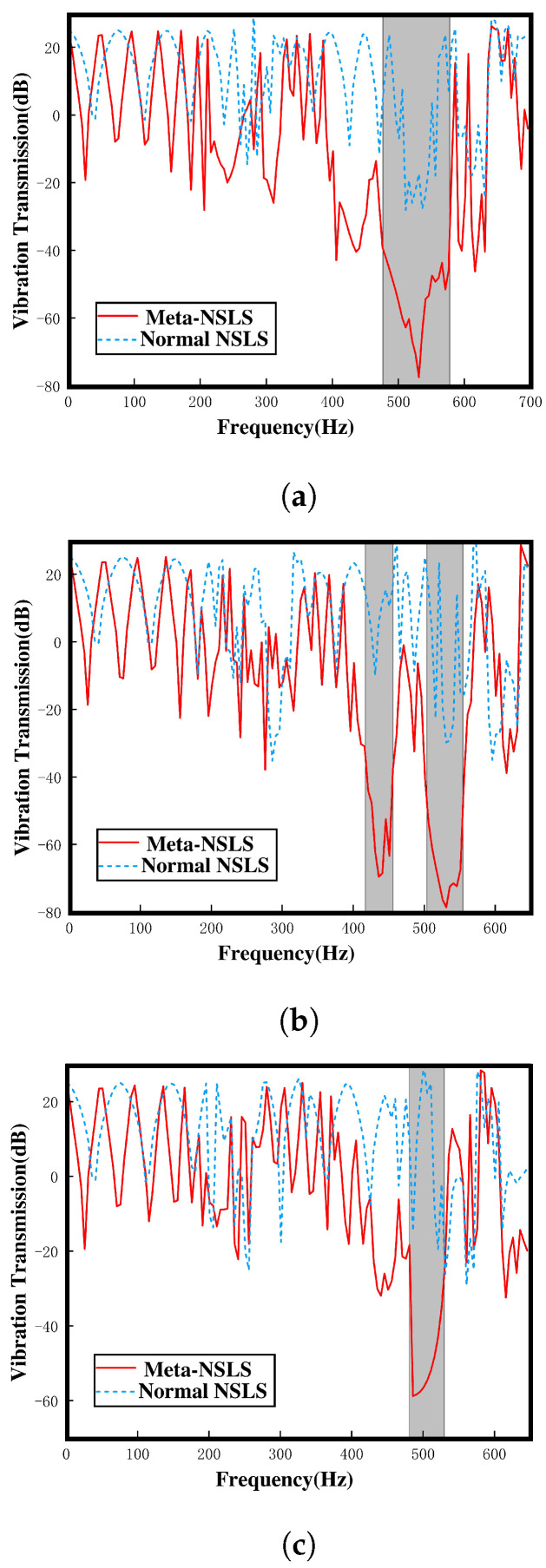
Vibration transmittance spectra of normal and meta-NSLS structures: (**a**) open configuration, (**b**) intermediate configuration, (**c**) closed configuration.

Figure 16 display the results of vibration transmission obtained through simulation for the open configuration, intermediate configuration, and closed configuration of both models, as follows: normal NSLS (blue dotted line) and meta-NSLS (solid red line). The shaded regions represent the complete bandgap regions identified in the dispersion spectrum. When a distinct attenuation range emerges within the transmission spectrum, it signifies that vibrations within this frequency range cannot traverse the lattice, substantiating the vibration-suppressing attributes of the finite lattice structure.

Upon scrutiny of the dispersion spectrum of the selected normal NSLS, it becomes evident that none of the three states exhibit a complete bandgap. This observation corroborates the findings illustrated in Figure 16. Across the three proposed stable states, normal NSLS offers minimal vibration attenuation capabilities. In stark contrast, meta-NSLS exhibits substantial vibration attenuation within the complete bandgap across all three configurations, aligning effectively with the bandgap identified on the dispersion spectrum. Moreover, the transmission loss remains notably high (>50 dB) throughout a significant portion of the bandgap. Specifically, within the open configuration, a complete bandgap is observed, with transmission loss reaching 77 dB near the frequency of 531 Hz. In the intermediate configuration, two relatively distinct complete bandgaps are discernible. Transitioning to the closed configuration, the vibration response drops rapidly, with a peak transmission loss of 58 dB at 491 Hz.

Evidently, meta-NSLS surpasses normal NSLS in its capacity to deliver vibration attenuation. Furthermore, the bandgap distribution and effective bandgap width it offers in various stable states exhibit differences. Additionally, the vibration attenuation range (>50 dB) obtained through numerical simulation aligns notably well with the bandgap range identified in the dispersion spectrum, demonstrating a high degree of consistency.

These findings underscore the efficacy of meta-NSLS in affording robust vibration suppression capabilities. Furthermore, the disparities in bandgap characteristics across stable configurations emphasize the adaptability of meta-NSLS to cater to specific engineering requirements.

**Figure 17:**
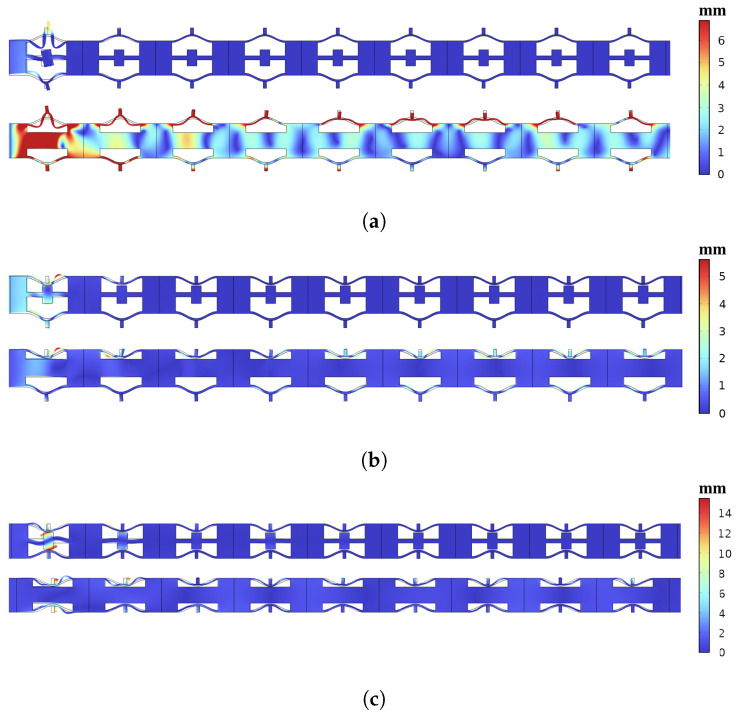
Dynamic responses of normal and meta-NSLS structures at (**a**) 531 Hz, (**b**) 519 Hz, (**c**) 486 Hz.

The dynamic response of the entire lattice structure is meticulously computed, as depicted in Figure 17, with the primary aim of visualizing and comparing the vibration damping performance between the two configurations. Specifically, for each of the three configurations, we selected representative frequencies situated within the bandgap range 531 Hz, 519 Hz, and 486 Hz. Notably, when juxtaposed with the normal NSLS (lower panel), the meta-NSLS (upper panel) exhibits a pronounced suppression of vibration transmission at frequencies within the bandgap. To illustrate further, taking Figure 17a as an illustrative example, when the excitation frequency (531 Hz) falls within the full bandgap range, the vibration energy within the meta-NSLS primarily localizes within the internal lumped mass resonator. Consequently, the excitation response is predominantly confined to the unit cells proximate to the excitation source, with minimal energy transfer occurring throughout the overall structure. In stark contrast, the normal NSLS exhibits a robust dynamic response. The upper and lower curved beam components of the normal NSLS, while akin to those of the meta-NSLS, similarly convey that, at this particular frequency, the vibrations within these components scarcely contribute to the overall vibration attenuation of the structure. This observation further corroborates that the local resonance of the designed meta-NSLS microstructure is the principal driver behind the effective vibration attenuation. In essence, the meta-NSLS design not only showcases its exceptional vibration damping performance but also substantiates the accuracy of the bandgap mechanism proposed in the preceding section.

## 5. Conclusions

This paper is dedicated to the research of NS mechanical metamaterials, introducing the concept of normal NSLS. Building upon this foundation, the study aims to retain the exceptional mechanical properties of NS mechanical metamaterials while evolving a novel meta-NSLS. NSLS is characterized by its unique physical property, allowing for the adjustment of stiffness and shape in response to external loads. Furthermore, the design process ensures controlled and orderly deformations. Notably, meta-NSLS exhibits remarkable features, including large deformability, reconfigurability, multi-directional bandgap tunability, and an extended range of tunable bandgaps. The study systematically investigates the dispersion relationships and bandgaps of both models, leading to several significant findings.

(1). Analysis of the mechanical properties of the proposed normal NSLS and meta-NSLS reveals their capacity to provide excellent energy absorption characteristics once suitable geometric parameters for the curved beams are determined. These configurations effectively reach various stable states during the compression process. Moreover, due to the complete identity of the curved beam sections in normal NSLS and meta-NSLS, there are minimal differences in their mechanical properties. Both models exhibit a fixed bending sequence during compressive deformation due to the differing thicknesses of the upper and lower curved beams. This enables rapid and efficient controllable and locally selectable deformations.
(2). Both configurations exhibit three stable states under the influence of external loads, namely the open configuration, intermediate configuration, and closed configuration, as proposed in this study. The selection of design parameters ensures that the stable deformation modes of each unit cell closely approach the elastic stability limit. Consequently, transitions between stable structures can be employed to adjust the frequency range of elastic wave attenuation. Notably, the bandgap distribution and effective bandgap width differ between different configurations. Importantly, meta-NSLS demonstrates superior bandgap capabilities compared to normal NSLS, offering significant deformability, reconfigurability, multi-directional bandgap tunability, and an extended range of tunable bandgaps. This suggests its potential for vibration isolation in practical applications.
(3). The study provides a detailed discussion of the influence of geometric parameters on the bandgap of meta-NSLS. The band structure under different deformation conditions and the influence of parameters on the bandgap are analyzed by numerical simulations. As the primary parameters ($l_{3}$, $h_{1}$, $h_{4}$) vary within reasonable ranges, the bandgap widths in the three configurations exhibit systematic changes. This observation underscores the sensitivity of bandgap properties to alterations in these key parameters, highlighting the crucial role of geometric adjustments in tailoring the bandgap characteristics of NS mechanical metamaterials.
4). Through the implementation of transmittance analysis and the presentation of dynamic response visualizations, the distinctions in vibration damping performance between the two structures are effectively elucidated. This serves to validate the precision of the bandgap mechanism delineated in the preceding section while substantiating that the local resonance of meta-NSLS is the primary factor in vibration suppression. These discoveries underscore the potency of meta-NSLS in delivering robust vibration suppression capabilities. Additionally, the variations in bandgap features among stable configurations ensure the adaptability of meta-NSLS to cater to precise engineering demands.

In summary, as a novel 3D-printed structure with internal resonance characteristics and tunable bandgap properties, the meta-NSLS proposed in this article exhibits remarkable large deformability and the capability to realize multiple complete bandgaps in certain configurations. Furthermore, it demonstrates multi-directional tunability and broadening of bandgaps through the transition between different configurations. These characteristics hold great promise for practical engineering applications, including aerospace engineering, civil engineering, and more. In practical use cases, structures can be adjusted to attain the required deformation state for effective vibration isolation and reduction in accordance with the desired isolation wave frequency.

## Figures and Tables

**Table 1:** Geometric parameters for the two configurations (unit: mm).

Parameters	NSLS	Meta-NSLS
*L*	50	50
$l_{1}$	4	4
$l_{2}$	20	20
$t_{1}$	2	2
$t_{2}$	2.5	2.5
*H*	8	8
$h_{1}$	30	30
$h_{2}$	9	9
$h_{3}$	8	8
$h_{4}$		10

**Table 2:** Material properties.

	Mass Density (kg/m${}^{3}$)	Young’s Modulus (MPa)	Poisson’s Ratio
TPU	1280	24.4	0.25

**Figure A2:**
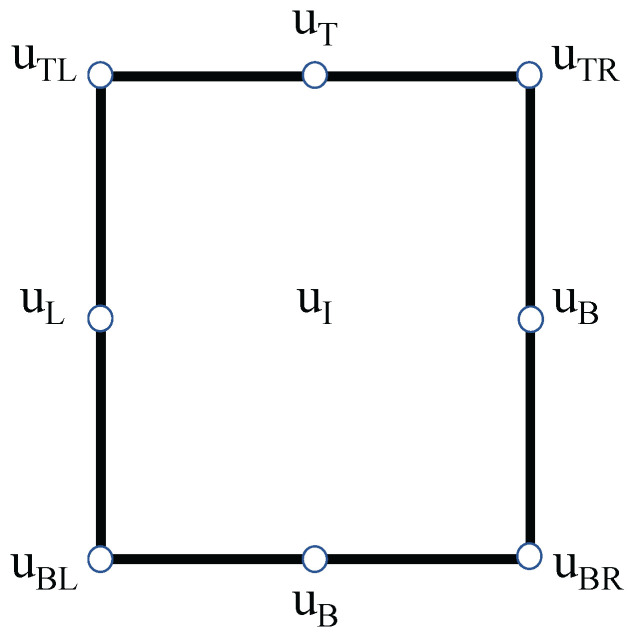
Regions considered to impose periodicity in the unit cell.

## Data Availability

Data are contained within the article.

## Appendix A. Dispersion Analysis

Taking the open configuration of a normal NSLS unit cell as an example, direct lattice vectors are defined on its unit cell, denoted as $a_{1}$ and $a_{2}$, as shown in Figure A1a. A reciprocal cubic lattice can be defined as shown in Figure A1b, with reciprocal lattice vectors denoted as $b_{1}$ and $b_{2}$. Vector $b_{1}$ is perpendicular to $a_{2}$, with a magnitude equal to $2\pi /a_{1}$, while $b_{2}$ is defined as a vector perpendicular to $a_{1}$, with a magnitude equal to $2\pi /a_{2}$.

Simultaneously, as shown in Figure A1b, the vertices of the region defined by IBZ are represented as Γ, *X*, *M*, and *Y*, with the wave domains of IBZ being Γ ($k_{x}$ = 0, $k_{y}$ = 0), *X* ($k_{x}$ = $\pi /a_{1}$, $k_{y}$ = 0), *M* ($k_{x}$ = $\pi /a_{1}$, $k_{y}$ = $\pi /a_{2}$), *Y* ($k_{x}$ = 0, $k_{y}$ = $\pi /a_{2}$). The wave vector *k* can be defined as a closed path along the perimeters defined by these vertices. This path is also indicated by arrows in Figure A1b, starting at Γ, passing through *X*, *M*, and *Y*, and eventually returning to Γ. Since the unit cell has spatial periodicity, according to Floquet–Bloch wave theory, the periodic displacement vector U(r) in reciprocal space is provided by

(A1) $$U\left(r\right)=U_{r}e^{i(\omega t-k\cdot r)}$$

where $U_{r}$ is the amplitude of the wave, k is the wave vector, and *i* is the imaginary unit. The dynamic equations of motion for the unit cell of a periodic lattice can be expressed as

(A2) $$\left(K-\omega^{2}M\right)u=F$$

where K and M are stiffness and mass matrices of the unit cell, respectively. The generalized nodal displacement and force vectors are denoted by u and F, respectively. The corresponding natural frequency ω for different wave vectors k can be determined by solving this equation and obtaining its eigenvalues. Thus, the band structure of the NSLS can be computed. As shown in Figure A2, the nodal displacement vector u and the force vector F are partitioned into nine components, and Bloch boundary conditions can be imposed using the following relation:

(A3) $$\begin{array}{cc} \; & u_{T}=u_{B}e^{-ik_{y}a_{2}},\\ \; & u_{R}=u_{L}e^{-ik_{x}a_{1}},\\ \; & u_{BR}=u_{BL}e^{-ik_{y}a_{2}},\\ \; & u_{TL}=u_{BL}e^{-ik_{x}a_{1}},\\ \; & u_{TR}=u_{BL}e^{-i(k_{x}a_{1}+k_{y}a_{2})}. \end{array}$$

where $u_{L}$, $u_{R}$, $u_{B}$, $u_{T}$, and $u_{I}$ represent the displacement of the left, right, bottom, top, and inner nodes, respectively. However, these vectors do not include the vertices, which are separately represented by $u_{BL}$, $u_{BR}$, $u_{TL}$, and $u_{TR}$.

Using the periodic boundary condition provided in Equation (A3), the displacement vector u can be expressed in terms of the reduced degrees of freedom as

(A4) $$\tilde{u}=Tu$$

where

(A5) $$u={\left[\begin{array}{c} u_{L}^{T}\;u_{R}^{T}\;u_{B}^{T}\;u_{T}^{T}\;u_{BL}^{T}\;u_{TL}^{T}\;u_{BR}^{T}\;u_{TR}^{T}\;u_{I}^{T} \end{array}\right]}^{T}$$

(A6) $$\tilde{u}={\left[\begin{array}{c} u_{L}^{T}\;u_{B}^{T}\;u_{BL}^{T}\;u_{I}^{T} \end{array}\right]}^{T}$$

(A7) $$T=\left[\begin{array}{cccc} I & 0 & 0 & 0\\ Ie^{-ik_{x}a_{1}} & 0 & 0 & 0\\ 0 & I & 0 & 0\\ 0 & Ie^{-ik_{y}a_{2}} & 0 & 0\\ 0 & 0 & I & 0\\ 0 & 0 & Ie^{-ik_{y}a_{2}} & 0\\ 0 & 0 & Ie^{-iLk_{x}a_{1}} & 0\\ 0 & 0 & Ie^{-i(k_{x}a_{1}+k_{y}a_{2})} & 0\\ 0 & 0 & 0 & I \end{array}\right]$$

where I represents an appropriate order of identity matrix, $k_{x}$ and $k_{y}$ represent the Bloch wave vectors along the x and y directions, with *a* being the lattice constant, and T denoting the transformation matrix. Substituting Equation (A4) into Equation (A2) and multiplying the resulting equation by $T^{H}$, the reduced degree of freedom equation can be obtained through force balance:

(A8) $$\left(T^{H}KT-\omega^{2}T^{H}MT\right)u^{\prime }=F^{\prime }$$

or

(A9) $$\begin{array}{c} \left(K^{\prime }-\omega^{2}M^{\prime }\right)u^{\prime }=F^{\prime } \end{array}$$

For free wave propagation, we have F=0. Therefore, Equation (A9) can be rewritten as

(A10) $$\left(K^{\prime }-\omega^{2}M^{\prime }\right)\tilde{u}=0$$

The periodic mapping matrix T incorporates the wave vector $\left(k_{x},k_{y}\right)$ dependency into the problem. Consequently, the stiffness and mass matrices include Bloch wave vector terms ($k_{x},k_{y}$), distinguishing them from conventional stiffness and mass matrices. To identify the characteristic frequencies associated with a specific Bloch wave vector ($k_{x},k_{y}$), we can formulate and solve the corresponding eigenvalue problem.

Typically, this can be achieved using numerical methods such as the finite element method (FEM), finite difference method (FDM), or boundary element method (BEM). These methods facilitate the computation of stiffness and mass matrices, after which a standard eigenvalue solver can be employed to address the eigenvalue problem. Upon solving, we can obtain a series of characteristic frequencies and mode shapes.

Considering *m* eigenvalues as functions of the wave vector and arranging them along the path Γ−X−M−Y−Γ, we can plot a band diagram. This diagram illustrates the NSLS or meta-NSLS’s dynamic response as a function of *k* and reveals the wavelengths that can propagate within the unit cell.
